# Herbal Preparation (Bromelain, Papain, Curcuma, Black Pepper) Enhances Mineralization and Reduces Glucocorticoid-Induced Osteoporosis in Zebrafish

**DOI:** 10.3390/antiox10121987

**Published:** 2021-12-14

**Authors:** Marta Carnovali, Gina Ramoni, Giuseppe Banfi, Massimo Mariotti

**Affiliations:** 1 IRCCS Orthopedic Institute Galeazzi, Via R. Galeazzi 4, 20161 Milan, Italy; marta.carnovali@grupposandonato.it; 2 Department of Biomedical, Surgical and Dental Sciences, University of Milan, Via Commenda 10, 20122 Milan, Italy; gina.ramoni@studenti.unimi.it (G.R.); banfi.giuseppe@hsr.it (G.B.); 3 School of Medicine, Vita-Salute San Raffaele University, Via Olgettina 58, 20132 Milan, Italy

**Keywords:** zebrafish, curcuma, osteoporosis

## Abstract

Natural foods with antioxidant properties, such as curcuma, papain, bromelain and black pepper, have been indicated as a potential natural therapeutic approach against osteoporosis. Zebrafish are an excellent animal model to study the effects of herbal preparations on osteogenesis and bone metabolism, both in physiological and in pathological conditions. Our study was aimed at evaluating whether curcuma-bromelain-papain-pepper herbal preparation (CHP) administered in embryos and adult fish is capable of promoting bone wellness in physiological and osteoporotic conditions. The effect of CHP has been studied in embryonic osteogenesis and glucocorticoid-induced osteoporosis (GIOP) in an adult fish model in which drug treatment induces a bone-loss phenotype in adult scales very similar to that which characterizes the bones of human patients. CHP prevented the onset of the osteoporotic phenotype in the scales of GIOP in adult zebrafish, with the osteoblastic and osteoclastic metabolic activity maintaining unaltered. CHP is also able to attenuate an already established GIOP phenotype, even if the alteration is in an advanced phase, partially restoring the normal balance of the bone markers alkaline phosphatase (ALP) and tartrate-resistant acid phosphatase (TRAP) and stimulating anabolic reparative processes. The results obtained indicated CHP as a potential integrative antioxidant therapy in human bone-loss diseases.

## 1. Introduction

The drugs used in the treatment of human bone diseases have shown relevant side effects [1]; for this reason, it is crucial to identify innovative non-invasive natural therapeutic approaches. A dietary intake of vegetables and fruits has an important impact on body wellness and, specifically, on bone health [2]. Indeed, nutraceuticals and herbal extracts, used for centuries in traditional medicine, have been included in numerous studies showing a crucial role in the prevention of human skeletal diseases because of their antioxidant and anti-inflammatory properties [3,4].

A good herbal preparation should be balanced, in terms of pharmacological functions. The main compound we have chosen for our preparation is curcumin, one of the most powerful sources of anti-inflammatory metabolites. Turmeric (*Curcuma longa* L.) is a perennial plant included in the ginger family, largely used in traditional Indian medicine. An extract from the turmeric rhizome, curcumin has potent anti-inflammatory, antioxidant, antiviral, antibacterial, antifungal and anticancer activities [5]. Unlike other anti-inflammatory agents, curcumin modulates inflammatory processes with a gradual multi-target action, without irritating gastric and intestinal walls and without causing unwanted effects under any circumstances [6].

The data available in the literature reported positive effects of curcumin on the skeletal system in in vitro and in vivo experimental models [7]. A study on rats shows that treatment with curcumin reverts bone changes resulting from ovariectomy, and therefore, it is effective as an estrogen therapy [8]. In a rat model of dexamethasone-induced osteoporosis, curcumin increases bone mineral density (BMD) and alkaline phosphatase (ALP), decreases carboxy-terminal collagen cross-links, increases mechanical strength and improves the trabecular microstructure of the bone, thus reducing dexamethasone-induced osteoporosis [9].

Bromelain is a set of proteolytic enzymes with a wide range of therapeutic benefits, including an anti-inflammatory activity that is also exploited for the treatment of disorders of the musculoskeletal system. We add bromelain in our preparation because it gives a specific antioxidant support against free radicals, different from curcumin [10].

The limit of curcumin is that it is not easily bioavailable because of its low intestinal adsorption. Piperine, the main constituent of *Piper nigrum* extract, increases intestinal absorption [11] and increase curcumin efficacy by inhibiting hepatic glucuronidation, a fundamental reaction for xenobiotic inactivation and excretion, thus ensuring better bioavailability of the other supplement molecules [12]. In addition, an in vitro study conducted on osteoclast precursors, in which it is administered in combination with curcumin, has shown that piperine is able to reduce the activity of osteoclasts and increase the inhibition of RANKL-induced osteoclastogenesis [11].

Papain, a protease, contributes to the degradation of excess of curcumin in the liver and the inflammatory cytokines, which have a crucial role in bone health and osteoporosis. Used together, these components have great efficacy. Overall, the characteristics of these natural substances suggest the use of this novel herbal preparation, named Curcuma-Bromelain-Papain-Pepper Herbal Preparation (CHP) as a potential integrative therapy in bone wellness maintenance [13].

*Danio rerio* (zebrafish) is an elective model for translational studies on human bone diseases. Specifically, the zebrafish embryo is a very consolidated model to study developmental osteogenesis [14], while adult scale represents a very helpful read-out system due to their availability, number, easy handling and histological similarity to the human bone matrix [15,16] and with the same cell type and regulatory mechanisms of deposition and resorption of human bone tissue [17,18]. Prednisolone (PN) is known to induce a dose-dependent loss of bone matrix (glucocorticoid-induced osteoporosis, GIOP) that can be evaluated in zebrafish scales with histological and biochemical tests [19]. The zebrafish GIOP model represents a powerful model to test the effectiveness of natural preparations to counteract the onset of bone-loss phenotype in scales.

## 2. Materials and Methods

### 2.1. Ethics Statement

This experimentation has been done in the Zebrafish Laboratory (IRCCS R. Galeazzi, GSD Foundation, Milan, Italy) in accordance with the Italian and European guidelines on research practice (EU Directive 2010/63/EU). Zebrafish experimental protocols were approved by the Italian Ministry of Health (authorization No.742/2019-PR).

### 2.2. Animals

Adult zebrafish (*Danio rerio*) AB strains were maintained in a ZEBTEC© bench top system (Tecniplast) under standard conditions (Westerfield, 2007). For the embryos experiment, single mates of adult fish were crossed. During the treatments, both adult fish and embryos were maintained at 28 °C in E3 medium (5 mM NaCl, 0.17 mM KCl, 0.33 mM CaCl_2_, 0.33 mM MgSO_4_).

### 2.3. Herbal Preparation

The herbal preparation (CHP) used in this study is a galenic product from an authorized preparatory pharmacist (Bravi Farmacie, Leno (BS), Italy), and it is composed of different plant extracts mixed with the following dosage per 500 mg:

- Bromelain (1200 GDU/g) 215 mg
- *Curcuma longa* (extract, 95% total curcuminoids) 131.5 mg (125 mg)
- Papain (1:100) 125 mg
- *Piper Nigrum* (dry extract, 95% piperine) 1.05 mg (1 mg)

All concentration data of CHP expressed in molarity (M) are referred to the concentration of curcumin and contain the other molecules according to the proportions indicated above. All the dilutions made subsequently to obtain the final treatment concentration were made in E3.

### 2.4. Chemicals

Prednisolone (1-Dehydrohydrocortisone, PN) (Sigma-Aldrich, Milan, Italy) was initially dissolved in dimetilsulfoxide (DMSO), then diluted in E3 medium to a final concentration of 80 μM.

### 2.5. Embryo Treatments

After breeding, zebrafish embryos were washed for five minutes with a methylene blue solution (2 mL of 0.1% methylene blue in 1 L E3 medium) to suppress eventual fungal and mold outbreaks [20]. Health conditions of embryos were checked using a light stereomicroscope (Olympus SZX-ZB7) [21] and grown up to 4 days post-fertilization (dpf) in E3 medium in a dark incubator at 28 °C.

### 2.6. Embryo Histochemical Analysis

Embryos were treated with different CHP concentrations from 1 nM to 100 μM and from 4 dpf to 7 dpf. Treatment solutions were changed every 24 h, and the viability rate was calculated at the end of the treatment. Embryos were euthanized by hypothermal shock [22], fixed in 3.5% formaldehyde 0.1 M sodium phosphate buffer solution and processed to evaluate osteogenesis using a two-color acid-free staining [23], Alcian blue 8GX (Sigma Aldrich) and Alizarin Red S (ARS, Sigma Aldrich), respectively. The embryo osteogenesis level was quantified for every single embryo analyzed under a light stereomicroscope (Olympus SZX-ZB7), and images were acquired using a discovery CH30 camera (TiEsseLab). The number of mineralized vertebral bodies was normalized for the body length (N.V./L.).

### 2.7. Adult Treatments and Scales Collection

Three different experiments were performed on adult fish. First, the 3-month-old young adult zebrafish were treated with 250 nM CHP for 14 days. Double staining with alizarin red/calcein was performed to quantify the growth rate of the mineralized tissue in the scales, as described below. The 9-month-old zebrafish were treated for 14 days with 500 nM CHP with/without 80 μM PN to verify the protective effects of CHP in the glucocorticoid-induced osteoporosis (GIOP) model. Finally, the 9month-old zebrafish were treated with 80 μM PN for 14 days and were then treated for another 14 days with PN with/without 500 nM CHP to verify the CHP curative effects in the GIOP model. All treatment solutions were changed every 48 h. At the end of each treatment, the fish were anaesthetized with 0.16 mg/mL tricaine methanesulfonate [20]. The scales were removed using Dumont^®^ Stainless steel forceps (Sigma Aldrich) from both sides of the fish’s body under a light stereomicroscope (Olympus SZX-ZB7).

### 2.8. Bone Matrix Vital Staining

In order to evaluate the new mineralized tissue deposed on scales, fish were stained with two successive live stainings, according to the Kimmel et al. method [21]. At the beginning of the treatment, fish were live stained with 0.005% Alizarin Red S (Sigma Aldrich) solution overnight in the dark at 28 °C, and at the end of the treatment, fish were stained with 0.005% calcein (Bis[*N*,*N*-bis(carboxymethyl)aminomethyl]fluorescein, Sigma Aldrich) E3 solution. Scales were carefully removed as previously described, fixed using 3.5% formaldehyde 0.1 M sodium phosphate buffer solution and then images were acquired using a fluorescence microscope (Olympus SZX-ZB7) with a Discovery CH30 camera (TiEsseLab, Milano, Italy).

### 2.9. Biochemical TRAP and ALP Assays in Scales

Biochemical TRAP and ALP activities were evaluated directly on explanted scales. Biochemical TRAP activity was performed as previously described by [17], while biochemical ALP activity was evaluated according to our method [18]. To perform both these analyses, the absorbance was read at 405 nm using a spectrophotometer (iMarkTM Microplate Reader, Bio-Rad, Milano, Italy).

### 2.10. Histological TRAP and ALP Assays in Scales

In each treatment, 5 fish were used and 10 scales were explanted for fish to obtain 50 scales for each treatment. A histological tartrate resistant acid phosphatase (TRAP) assay was performed using a Leukocytes Acid Phosphatase detection kit (Sigma Aldrich, Italy) on explanted scales following the manufacturer’s protocol; then, TRAP-positive scales were counted. A histological alkaline phosphatase (ALP) assay was done using a BCIP^®^/NBT liquid substrate (Sigma Aldrich, Italy) following the manufacturer’s protocol.

### 2.11. Statistics

Embryo data derived from 10 embryos treated for every CHP concentration performed in three independent experiments, which produced comparable results. Data were plotted on SigmaStat3.5 software and analyzed by a one-way analysis of variance (ANOVA), followed by a Dunn’s test for multiple comparisons. Data from adult fish experimentations were obtained by 10 scales explanted from 5 fish for each treatment group. Each experiment was repeated three times with similar results. The results were expressed as the mean of the means of the three independent experiments associated with the standard deviation versus the control. The significance was determined for *p*-values by using an ANOVA and Bonferroni test for multiple comparisons. All the significance values were set at *p* < 0.05 (*), *p* < 0.01 (**) and *p* < 0.001 (***).

## 3. Results

### 3.1. CHP Treatment Stimulates Osteogenesis in Zebrafish Embryos

**Figure 1:**
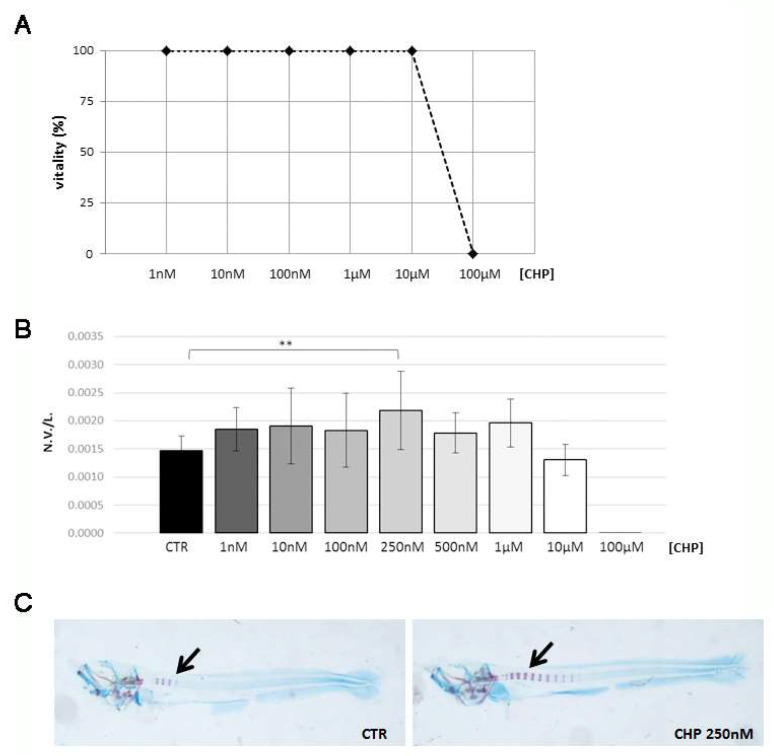
(**A**) Viability curve of zebrafish embryos evaluated at the end of the treatment at different CHP concentrations. (**B**) Mineralization rate of embryos treated with different concentrations of CHP with respect to the untreated control (CTR), assessed as the ratio of number of vertebrae positive to alizarin red and length of embryo in micrometers (N.V./L.) (CTR vs. 250 nM CHP, **, *p* < 0.01). (**C**) Double staining alcian-blue/alizarin-red on untreated embryos (CTR, left) and treated with 250 nM CHP (right), which gave evidence for the difference of mineralization in the vertebrae, stained in violet (black arrow).

A toxicity study was performed by exposing embryos (4 dpf to 7 dpf) to different concentrations of CHP, from 1 nM to 100 μM, and the viability rate was calculated at the end of the treatment. The 10µM concentration of CHP represented the vitality threshold for embryos since all the embryos were found dead at 100 μM CHP (Figure 1A).

Next, we assessed the effects of CHP on embryonic osteogenesis through a double histochemical staining with alcian blue and alizarin red on embryos exposed to different concentrations of CHP, from 1 nM to 100 μM. By calculation of the ratio between the number of vertebrae displayed and the length of the larva measured in micrometers (N.V./L.), it a statistically significant increase of 49% in the rate of osteogenesis at 250 nM was shown with respect to the untreated control (Figure 1B). Figure 1C shows the increase of the number of mineralized vertebrae in 250 nM CHP-treated embryos with respect to controls.

### 3.2. CHP Stimulates the Deposition of New Mineralized Matrix in Juvenile Zebrafish Scale

**Figure 2:**
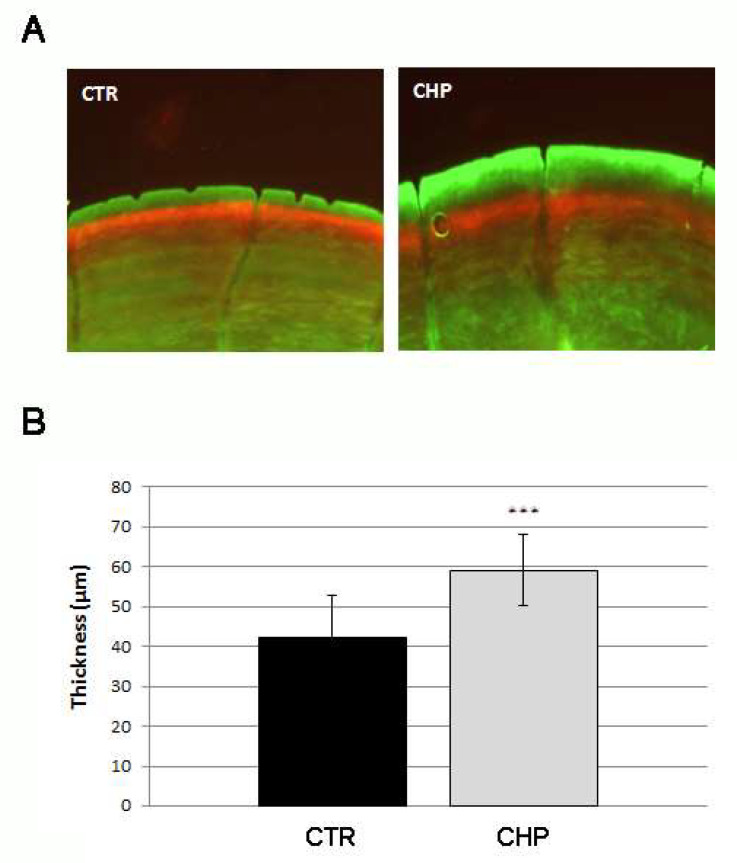
(**A**) Double staining alizarin red/calcein highlights the growth rate of the mineralized tissue in the scale; the growth ring of the treated fish (CHP) was significantly thicker than that of the untreated control (CTR). (**B**) Growth rate evaluation of a scale by measuring the thickness of the new ring in micrometers. The thickness in treated fish (CHP) was 40% greater than in controls (CTR) (CHP vs. CTR, ***, *p* < 0.001).

The concentration of 250 mM (effective on embryonic osteogenesis) was chosen for the following experiments on juvenile fish. A double staining with alizarin red and with calcein was performed to evaluate the deposition rate of new mineralized matrix during scale growth in the presence of CHP. The herbal preparation was administered to juvenile fish (3 months old) through exposure for 14 days at 250 nM in E3. The deposition process of new mineralized matrix in the scale was quantified by measuring the thickness of the new ring, marked in green fluorescence, of treated fish with respect to the controls (Figure 2A). The growth ring of CHP-treated scales, measured in micrometers, was found to be 40% greater than untreated ones (CHP vs. CTR, *p* < 0.001) (Figure 2B). The treatment with CHP was able to accelerate the growth process of the scale ring.

### 3.3. Protective Effect of CHP on PN-Dependent Osteoporotic Phenotype in Adult Fish Scales

**Figure 3:**
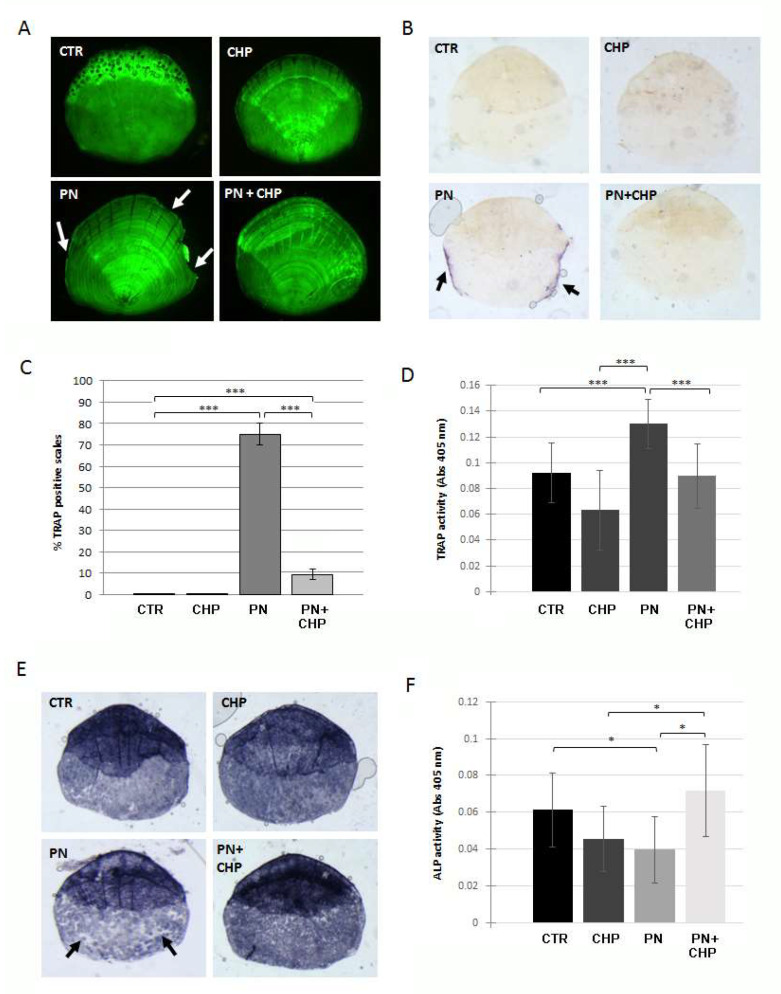
(**A**) Morphological analysis of the individual scales stained with calcein. The scales treated with PN show resorption lacunae along the borders (white arrows), while the scales treated with CHP or with PN (PN + CHP) were comparable to untreated controls (CTR). (**B**) Histochemical staining for TRAP activity on PN, CHP and PN + CHP scales. A TRAP-positive signal was strongly detected along the scale border of PN-treated fish, surrounding a bone resorption area. (**C**) A quantification of TRAP-positive scales indicated that 75% of PN-treated scales were found resorbed, while there was only 9% after co-treatment PN + CHP (CTR vs. PN, ***, *p* < 0.001; CTR vs. PN + CHP, ***, *p* < 0.001; PN vs. PN + CHP, ***, *p* < 0.001). (**D**) TRAP activity, measured by biochemical assay and normalized for scale area, confirmed high resorbing activity in PN-treated scales, neutralized by co-treatment PN + CHP (CTR vs. PN, ***, *p* < 0.001; CHP vs. PN, ***, *p* < 0.001; PN vs. PN + CHP, ***, *p* < 0.001). (**E**) A histological assay of ALP activity was performed on individual scales. The scales of the fish treated with PN showed large white areas where the ALP activity was missing (black arrows), while the scales of the fish treated with only CHP and PN + CHP showed ALP activity comparable to the CTR. (**F**) Biochemical assay of ALP activity. The ALP activity in the scales of the fish treated with prednisolone (PN) was significantly lower than the untreated control (CTR), while the addition of CHP prevented ALP activity downregulation (PN vs. CTR, *, *p* < 0.05; PN vs. PN + CHP, *, *p* < 0.05; CHP vs. PN + CHP, *, *p* < 0.05).

We investigated the preventive anti-osteoporotic effect of CHP on a glucocorticoid-induced osteoporosis model on zebrafish scales. Adult fish were treated for 14 days with 500 nM CHP alone or in combination with80 μM PN, and the integrity of the mineral matrix in the scales was evaluated by calcein staining. There was evidence for resorption lacunae at the scale borders after the treatment with PN, while the simultaneous treatment with CHP avoided the onset of the bone-loss phenotype (Figure 3A). After the treatment with CHP only, no resorption lacunae were visible.

To evaluate the osteoclast resorption activity, the histochemical TRAP assay was performed on scales treated or untreated with PN and CHP. As shown in Figure 3B, scales from fish treated with PN showed intense TRAP activity, while fish also treated with 500 nM CHP completely prevented osteoclast activation. The percentage of TRAP-positive scales was reduced to 9% in PN + CHP treated scales compared to the 75% positive scales after PN alone (Figure 3C).

To measure the resorption activity, we used a TRAP biochemical assay, confirming that a 500 nM CHP treatment inhibited the increase of TRAP in zebrafish scales after PN treatment (Figure 3D). A decreased ALP osteoblast activity could be detected in the scales of adult fish after PN treatment (Pasqualetti et al., 2015). PN reduced ALP activity in all the fish scales, whereas a simultaneous treatment with 500 nM CHP prevented such reduction (Figure 3E). The loss of ALP signal in PN-treated scales was extended to the entire surface of the scale, and the incubation of 500 nM CHP together with PN restored the whole signal (Figure 3F).

### 3.4. CHP Showed a Curative Effect on PN-Induced Bone Loss in Adult Fish Scales

We investigated the therapeutic effect of CHP on a glucocorticoid-induced osteoporosis model on zebrafish scales. Adult fish were treated with 80 μM PN for 14 days and, next, were treated or untreated with 500 nM CHP for an additional 14 days.

**Figure 4:**
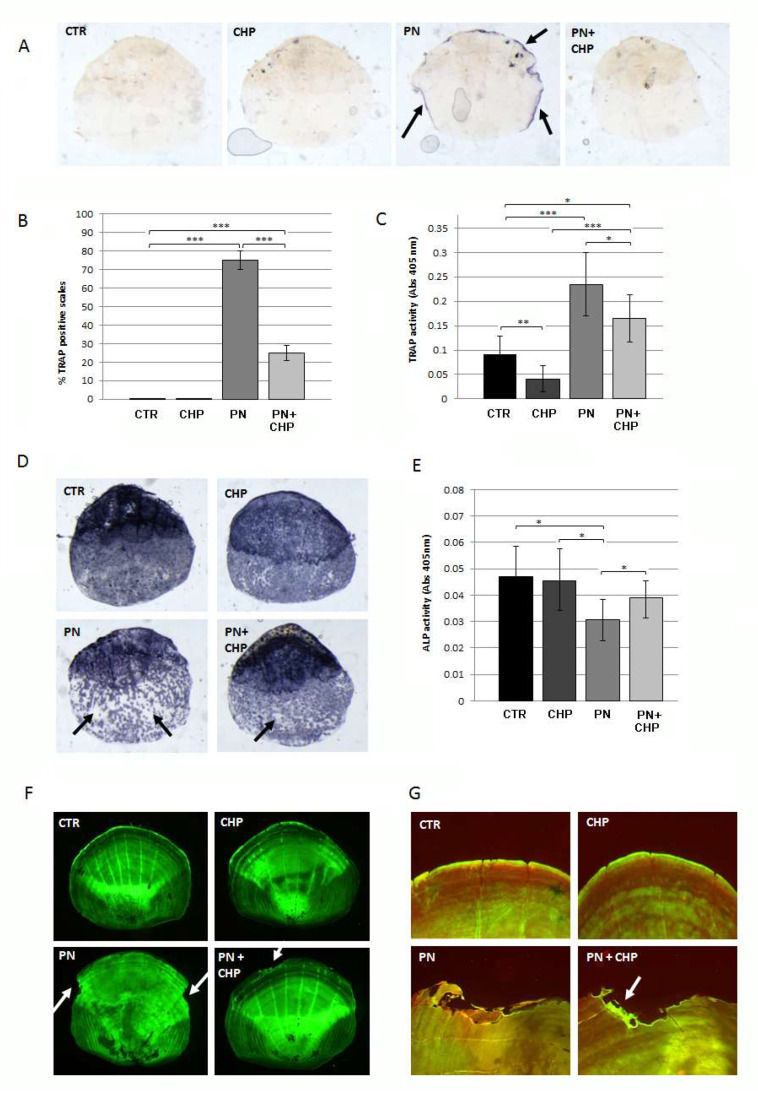
(**A**) Histochemical staining for TRAP activity on PN, CHP and PN + CHP scales. A TRAP-positive signal was strongly detected along the scale border of PN-treated fish, surrounding a bone resorption area. (**B**) A quantification of TRAP-positive scales indicated that 75% of PN-treated scales were found resorbed compared with 25% after co-treatment PN + CHP (CTR vs. PN, ***, *p* <, 0.001; CTR vs. PN + CHP, ***, *p* < 0.001; PN vs. PN + CHP, ***, *p* < 0.001). (**C**) TRAP activity, measured by biochemical assay and normalized for scale area, confirmed high resorbing activity in PN-treated scales, later neutralized by treatment with CHP (CTR vs. CHP, ** *p* < 0.01; CTR vs. PN, ***, *p* < 0.001; CTR vs. PN + CHP, *, *p* < 0.05; CHP vs. PN, ***, *p* < 0.001; CHP vs. PN + CHP, *, *p* < 0.05; PN vs. PN + CHP, *, *p* < 0.05). (**D**) Histological assay of ALP activity performed on individual scales. The scales of the fish treated with PN showed large areas without ALP activity (black arrows), while the scales of the fish treated with PN + CHP partially restored ALP activity if compared with CTR and CHP alone. (**E**) Biochemical assay of ALP activity. The ALP activity in the scales of the fish treated with prednisolone (PN) was significantly lower than the untreated control (CTR) (PN vs. CTR, *p* < 0.05) and CHP alone (CHP vs. PN, *p* < 0.05), while the addition of CHP (PN + CHP) partially restored ALP activity (PN + CHP vs. PN, *p* < 0.05). (**F**) Morphological analysis with calcein showed very large areas of resorption in PN-treated scales (PN), while they appeared drastically reduced after treatment with CHP (PN + CHP). No resorbed borders could be detected in negative controls (CTR and CHP alone). (**G**) Alizarin-calcein double staining revealed the presence of new mineralized matrix deposition only in CHP-treated scales (PN + CHP), with respect to untreated controls (PN). No resorbed areas could be detected in negative controls (CTR and CHP alone).

To evaluate the osteoclast resorption activity, the histochemical TRAP assay was performed on scales treated or untreated with CHP after PN. As shown in Figure 4A, scales from fish treated with PN showed intense TRAP activity, while fish treated later with 500 nM CHP were able to suppress osteoclast activation in scales. The percentage of TRAP-positive scales was reduced from 75%, in PN-treated scales, to 25% after PN + CHP incubation (Figure 4B).

A TRAP biochemical assay confirmed that the addition of 500 nM CHP reduced the PN-dependent increase of TRAP in zebrafish scales but did not completely restore the physiological state as in controls (Figure 4C).

The analysis of ALP activity in the scales confirmed that the administration of CHP to a PN-induced bone-loss phenotype has significant beneficial effects associated with increased ALP activity but still does not reach CTR levels, as demonstrated by histological (Figure 4D) and biochemical (Figure 4E) evaluation.

In addition, calcein staining was used to evaluate the quality of the mineral matrix in the scales. Resorption lacunae on the scale borders were detected after treatment with PN, while there was evidence for a reduction of number and extension of resorbing areas after treatment with CHP (Figure 4F). The analysis of the bone matrix deposition by alizarin-calcein double staining showed that new mineralized matrix was synthesized in the lacuna area only after treatment with CHP (Figure 4G), suggesting an activation of bone anabolic activity.

## 4. Discussion

The zebrafish embryonic stage is an elective model for osteogenesis studies, while the adult model has growing importance for the study of bone metabolism and remodeling [15]. In adult fish, it is possible to model human bone pathologies, such as secondary osteoporosis, while the scale represents an innovative, informative and ethically correct read-out system [19]. Given these characteristics, zebrafish represent an ideal model to study the effects of phyto-preparations, such as CHP, on bone metabolism in vivo.

CHP has been made using *Curcuma longa*, *Piper nigrum*, bromelain and papain. Curcumin, a component of *Curcuma longa* extract, has powerful anti-inflammatory and antioxidant activities [24], and it has important molecular targets that participate in the regulation of bone metabolism and remodeling [25,26]. Nevertheless, low bioavailability of curcumin is a great limitation for clinical development [27]. Piperine, the main constituent of black pepper extract, is a potent inhibitor of intestinal and hepatic glucuronidation and greatly enhances the bioavailability of curcumin after intestinal absorption [28]. Distribution studies showed its preferential accumulation in the liver, where it may exert toxic effects. Hepatic over-loading of curcumin can be avoided by using papain and bromelain as proteolytic enzymes, which break down proteins.

In this study, CHP was initially used on the embryonic zebrafish model by evaluating its toxicity level. This toxicity is not detected in other animal models because most of the curcumin that is administered orally, for example in the rat, is excreted in the feces and urine. On the contrary, the zebrafish embryo is unable to metabolize and eliminate curcumin, which therefore accumulates in the yolk sac and skin cells, leading to the death of the embryo. It is interesting to note how our studies show a lethal concentration of 100 μM, while a previous study defined 7.5 μM as the lethal dose of curcumin only on zebrafish embryo [29]. Another study on zebrafish embryos identified LD50 as 135 μg/mL (350 μM), but an early mortality window appeared at 30–50 μg/mL (50–150 μM) curcumin [30].

Different curcumin extract methods may modulate the proportion of single components and modify the toxicity threshold. In our study, the main difference was probably due to a protective role of bromelain, papain and piperine.

Once the toxicity thresholds were defined, the effects of CHP on the embryonic osteogenesis were evaluated. Embryo treatment with a 250 nM CHP concentration resulted in a statistically significant difference, where the ratio between the number of vertebrae and the length of the embryo was greater than 50% compared to the control.

A positive effect on bone mineralization was also found in young adult zebrafish, where we showed a deposition of mineralized matrix in the scale growth ring 40% higher than in the control, treating the animals with the same concentration of CHP that was able to significantly increase embryonic osteogenesis.

It has been reported that curcumin stimulates anabolic processes in bone in vitro and in vivo. It has been found to promote osteoblast differentiation in vitro [31,32] and enhances the ability of mesenchymal stem cells to differentiate in skeletal cells [33,34].

Undifferentiated mesenchymal-like stem cells play a role in zebrafish embryos during skeletogenesis [35] and in adult fish in a growing ring of scales [36]; stem cell population could be the target of stimulatory effects of curcumin.

Having obtained significant results in the two physiological models of the embryo and adult, we verified whether CHP had effects in the treatment of pathological conditions related to the bone skeleton. We focused on an adult glucocorticoid-induced osteoporosis (GIOP) model to investigate the protective and/or curative effects of CHP on the bone-loss phenotype using scales as a readout system. 

In the investigation of the protective effects of CHP in the GIOP model, we demonstrated that CHP was able to effectively counteract the negative effects of glucocorticoids on bone tissue, preventing the establishment of the osteoporotic phenotype in the scales.

Data in the literature showed how preventive incubation with curcumin in dexamethasone-treated osteoblast in vitro stimulated the resistance to apoptosis [9]. Another study examined the effects of co-treatment with curcumin in the GIOP model in mice, demonstrating that it is able to increase the levels of bone formation markers, such as osteocalcin, and in parallel, to decrease bone resorption markers, such as TRAP and cathepsin K [37].

Black pepper extract has never been tested in GIOP models, but in a rat periodontitis study, piperine was able to decrease alveolar bone loss and reform trabecular microstructure [38]. Although bromelain has important antioxidant properties, it has never been tested in bone models in vitro or in vivo, much less in pathological models.

The experiments on the GIOP model used CHP concentrations equal to 500 nM, while the concentration used in young adults was 250 nM. The difference was mainly due to the fact that in 9-month-old fish used in the GIOP model, the 250 nM CHP concentration was not effective (data not shown). By increasing the dose to 500 nM, we found the effects previously described. Our hypothesis can be that, as for all animals, the administration of the quantities of a drug must be formulated based on the weight of the animal. The size of the 9-month-old fish compared to the 3-month-old was approximately double, both in weight and length.

In evaluating the curative effects of CHP on the GIOP model, we confirmed a positive effect on the bone turnover imbalance established by PN. The data show a partial recovery of physiological enzymatic activity, assuming that it may take longer to completely inhibit the pathogenic stimulus triggered by PN.

The analysis with the double staining of alizarin red and calcein showed the persistence of the reabsorption gaps, even after 14 days of treatment with PN and CHP. However, a weak signal of deposition of a new mineralized matrix was detectable on the edge of the gap, indicating that the resorbing activity gave way to an initial attempt to repair the bone matrix aimed at closing the gap. These data support the hypothesis that CHP needs longer treatments to completely reverse the PN-induced osteoporotic phenotype in zebrafish scales.

Data in the literature showed how curcumin administered in a dexamethasone-induced osteoporosis model in rats is able to increase BMD and ALP activity, improve bone microstructure and increase mechanical strength, reducing the osteoporotic phenotype overall [9].

In human bone-loss diseases, such as osteoporosis, the oxidative and inflammatory components play a fundamental role [39].

The discovery of natural extracts or compounds with beneficial effects on osteoporosis is opening a new road toward a sustainable approach to the disease [40]. Most of them specifically exhibit antioxidant and anti-inflammatory properties, which attenuate the bone-loss phenotype in vivo [41].

Curcumin is known for its antioxidant effects and is capable of modulating a number of molecular targets, including transcription factors that are important for the protection of osteoblasts [42]. In addition, curcumin suppressed in vivo osteoclast formation by increasing antioxidant activity and modulating RANKL signaling, as reported by studies on ovariectomized mice [43] and a hind-limb suspension microgravity model [44].

The combination of curcumin with other active ingredients, such as black pepper, papain and bromelain, can increase the antioxidant potential and the bioavailability of individuals, thus increasing their effectiveness as a therapeutic agent on bone diseases. Piperine has been used in in vitro studies in murine macrophages and human CD14+ monocytes, where it inhibits the formation of TRAP-positive multinucleated osteoclasts [45]. Piperine also increases intestinal absorption [11] and inhibits hepatic glucuronidation, a fundamental reaction for the inactivation and excretion of xenobiotics, thus ensuring better bioavailability of the other supplement molecules [12]. In addition, an in vitro study conducted on osteoclast precursors in which it is administered in combination with curcumin has shown that piperine is able to reduce the activity of osteoclasts and increase the inhibition of RANKL-induced osteoclastogenesis [11]. Bromelain is a set of proteolytic enzymes with a wide range of therapeutic benefits, including an antioxidant activity in vitro [46] that is also exploited for the treatment of disorders of the musculoskeletal system [10]. Papain, another proteolytic enzyme, derived from papaya, exerts hepatic detox activity in concert with bromelain. In addition, papaya is known to possess antioxidant properties helpful in osteoporosis treatment [47].

## 5. Conclusions

In this study, the zebrafish confirms itself as an optimum model for the study of bone metabolism in the read-out embryonal model of osteogenesis, as well as an adult osteoporosis scale model. Moreover, the zebrafish is a fast, excellent model to test natural molecules and extracts and can be very useful to contribute to the study of alternative natural therapies.

Overall, the characteristics of the natural substances that we have chosen for CHP, curcumin, bromelain, piperine and papain lead to a natural preparation with synergic characteristics and great potential as a potential integrative antioxidant therapy in human bone-loss diseases.

## Data Availability

Data is contained within the article.
